# HIV co-opts a cellular antiviral mechanism, activation of stress kinase PKR by its RNA, to enable splicing of *rev/tat* mRNA

**DOI:** 10.1186/s13578-023-00972-1

**Published:** 2023-02-11

**Authors:** Lise Sarah Namer, Alex Harwig, Stephan P. Heynen, Atze T. Das, Ben Berkhout, Raymond Kaempfer

**Affiliations:** 1 Department of Biochemistry and Molecular Biology, Institute of Medical Research Israel-Canada, The Hebrew University-Hadassah Medical School, 9112102 Jerusalem, Israel; 2 Laboratory of Experimental Virology, Department of Medical Microbiology, Amsterdam UMC, 1105 AZ Amsterdam, The Netherlands

**Keywords:** Cellular antiviral response, mRNA splicing, HIV, Viral RNA, PKR activation, eIF2alpha phosphorylation, RNA pseudoknot, Phylogenetic conservation of RNA structure

## Abstract

**Background:**

Activation of RNA-dependent stress kinase PKR, especially by viral double-stranded RNA, induces eukaryotic initiation factor 2 α-chain (eIF2α) phosphorylation, attenuating thereby translation. We report that this RNA-mediated negative control mechanism, considered a cornerstone of the cell’s antiviral response, positively regulates splicing of a viral mRNA.

**Results:**

Excision of the large human immunodeficiency virus (HIV) *rev/tat* intron depends strictly on activation of PKR by the viral RNA and on eIF2α phosphorylation. *Rev/tat* mRNA splicing was blocked by viral PKR antagonists Vaccinia E3L and Ebola VP35, as well as by a *trans*-dominant negative mutant of PKR, yet enhanced by overexpressing PKR. Expression of non-phosphorylatable mutant eIF2αS51A, but not of wild type eIF2α, abrogated efficient splicing of *rev/tat* mRNA. By contrast, expression of eIF2αS51D, a phosphomimetic mutant of eIF2α, left *rev/tat* mRNA splicing intact. Unlike eIF2αS51A, eIF2αS51D does not inhibit eIF2α phosphorylation by activated PKR. All HIV mRNA species contain terminal *trans*-activation response (TAR) stem-loop sequences that potentially could activate PKR, yet even upon TAR deletion, HIV mRNA production remained sensitive to inhibitors of PKR activation. Bioinformatic and mutational analyses revealed a compact RNA pseudoknot upstream of 3′-terminal TAR that promotes splicing by activating PKR. Supporting its essential role in control of splicing, this pseudoknot is conserved among diverse HIV and nonhuman primate SIVcpz isolates. The pseudoknot and 3′-terminal TAR collaborate in mediating PKR-regulated splicing of *rev/tat* intron, the pseudoknot being dominant.

**Conclusions:**

Our results on HIV provide the first example of a virus co-opting activation of PKR by its RNA, a cellular antiviral mechanism, to promote splicing. They raise the question whether other viruses may use local activation of host kinase PKR through RNA elements within their genome to achieve efficient splicing of their mRNA. Our experiments reveal an indispensable role for eIF2α phosphorylation in HIV *rev/tat* mRNA splicing that accounts for the need for PKR activation.

**Supplementary Information:**

The online version contains supplementary material available at 10.1186/s13578-023-00972-1.

## Background

Stress induces a reversible phosphorylation of the eukaryotic initiation factor 2 α-chain (eIF2α) [1, 2], blocking GDP/GTP exchange needed for recycling of eIF2 between rounds of protein synthesis [3]. A prominent eIF2α kinase is PKR, an RNA-dependent serine/threonine protein kinase expressed in latent form in most cells. PKR plays an essential role in the interferon (IFN)-mediated antiviral response. Once activated by double-stranded RNA, PKR phosphorylates eIF2α and thereby inhibits mRNA translation, blocking virus spread and inducing apoptosis of infected cells [4, 5]. To enable *trans*-autophosphorylation needed for kinase activation, PKR must transiently form a homodimer on the activating RNA [6, 7]. Binding and activation of PKR are related in a complex manner. Stable binding of RNA to PKR can inhibit its activation, emphasizing the need for transient binding. Thus, by binding tightly to PKR, adenovirus VA RNA sequesters PKR in an inactive state, to prevent kinase activation [8].

Viral double-stranded RNA is not the only inducer of PKR activation. Short intragenic RNA elements can control cellular gene expression at translation or splicing by strongly activating PKR and inducing eIF2α phosphorylation [9, 10]. Human *IFN-γ* mRNA activates PKR and uses this mechanism to attenuate its own translation through eIF2α phosphorylation, thus preventing overexpression; the *IFN-γ* RNA activator of PKR is a pseudoknot that spans the 5’-untranslated region (5′-UTR) and first 26 codons and undergoes dynamic refolding upon ribosome passage [9, 11]. While the canonical role of activated PKR is to control protein synthesis, splicing of *tumor necrosis factor-*α (*TNF-α*) pre-mRNA depends on its ability to activate PKR [10, 12, 13]. A *cis*-acting RNA element within the 3′-UTR of *TNF-α* pre-mRNA, containing a pseudoknot, potently activates PKR, to render mRNA splicing highly efficient yet dependent on phosphorylation of eIF2α, without repressing translation [10, 13].

Control of mRNA splicing by PKR activation and eIF2α phosphorylation extends beyond the inflammatory response. Spliceosome assembly on human *globin* pre-mRNA and splicing depend strictly on activation of PKR by an intragenic activator element and on nuclear eIF2α phosphorylation [14]. Thus far, these are the only genes for which this control mechanism has been shown and it is exceptional, as witnessed by the fact that the closely related *TNF-β* gene, reflecting the majority of genes, lacks an RNA element that can activate PKR and its mRNA is spliced an order of magnitude less efficiently than *TNF-α* mRNA [10].

The *cis*-acting nature of the intragenic *TNF-α* RNA element [10] is accounted for by the fact that *trans*-autophosphorylation of PKR needed for its activation and eIF2α phosphorylation both are local, transitory events in the cell in close proximity to the activating RNA molecule, followed promptly by dephosphorylation [15–17]. Upon dephosphorylation, PKR returns to its inactive state while eIF2α can resume initiation of translation. Global levels of activated PKR and phosphorylated eIF2α in the cell are not affected. Mere insertion of the *cis*-acting *TNF-*α RNA element into the *TNF-β* gene sufficed to increase *TNF-β* mRNA splicing efficiency by an order of magnitude [10], showing that an intragenic RNA activator of PKR can locally render splicing far more efficient. Localized PKR activation accounts also for the *cis*-acting nature of the *IFN-γ* activator of PKR, which does not inhibit translation globally [9].

Activation of PKR by viral RNA and phosphorylation of eIF2α, a negative control mechanism that inhibits translation, is considered to be a cornerstone of the cell’s antiviral response. Here, we examined the hypothesis that a virus could subvert this cellular antiviral mechanism to its advantage. Specifically, we asked whether activation of PKR and eIF2α phosphorylation might control splicing of human immunodeficiency virus-1 (HIV) RNA. Considering that all HIV mRNA species contain at both termini a stem-loop, the *trans*-activation response (TAR) RNA element, that potentially could activate PKR [18, 19], we hypothesized that HIV mRNA splicing might depend on PKR activation by its RNA and on eIF2α phosphorylation. Our finding is that this is indeed the case. HIV-1 has co-opted this cellular antiviral mechanism for its own benefit.

## Methods

### Plasmid constructs

HIV-1 molecular clone pLAI [20], doxycycline-dependent derivatives HIV-rtTA-TARm [21] and HIV-rtTA-ΔTAR (ER2 variant) [22] were described previously. A plasmid expressing the 3′ half of HIV-1 (pcDNA-3’HIV) was generated by PCR using pLAI as template with primers RevAUGmut (5′ACCTAAGCTTGCAGGAAGAAGCGGAGACA) and pLAI-3′seq [22]. The resulting fragment was TA-cloned into pCR2.1-TOPO (Invitrogen) and insertion confirmed by DNA sequencing. The validated clone was digested with EcoRI and the fragment containing the 3’ half of HIV-1 inserted into the corresponding site of pcDNA3, allowing expression from the constitutive CMV promoter. Vectors expressing human *PKR* [13], *K296R trans*-dominant negative mutant PKR [23], *eIF2*α*S51A* and *eIF2*α*S51D* [9, 10, 24] were described previously. *eIF2*α*wt* expression vector was generated from *eIF2*α*S51A* DNA by PCR using phosphorylated DNA primers [10].

### Mutagenesis

To generate mutations in *rev/tat*, pcDNA-3′HIV DNA was used as template for PCR using phosphorylated mutant DNA primers and KOD polymerase (Novagen) to generate linear full-length plasmid DNA carrying blunt ends; the product was purified on a 1% agarose gel and self-ligated. Primer sequences will be supplied on request. DNA sequencing was used to verify the constructs.

### Transient transfection

For transfection with full-length HIV vectors, human embryonic kidney (HEK-293 T) cells were cultured as monolayer in Dulbecco’s modified Eagle’s medium (Invitrogen) supplemented with 10% fetal bovine serum (FBS), penicillin (100 U/ml) and streptomycin (100 μg/ml) at 37 °C and 5% CO_2_. Cells were cultured in 10-cm^2^ wells and transfected with 5 μg HIV plasmid DNA using Lipofectamine 2000 (Thermo Fisher Scientific), in the absence or presence of PKR inhibitor PKRi *{8-(imidazol-4-ylmethylene)-6H-azolidino(5,4-g)benzothiazol-7-one}*(C16, Calbiochem CAS 608512-97-6) [25, 26], *E3L* expressing plasmid pEF5-E3L [23], or empty vector pcDNA3; the total amount of pEF5-E3L and pcDNA3 was kept constant at 500 ng. Cells transfected with HIV-rtTA vectors TAR^m^ and ΔTAR were cultured in presence of 1 μg/ml doxycycline (dox; Sigma D-9891).

pcDNA-3′HIV DNA was transiently transfected into baby hamster kidney (BHK-21) cells, the cell line used to analyze PKR-dependent regulation of *IFN-γ*, *TNF-α* and *globin* genes [9, 10, 12, 14]. pEGFP-N3 DNA (Clontech)(0.5 μg) was included to assess transfection efficiency. BHK-21 cells cultured in 2 ml DMEM medium containing 10 mmol HEPES pH 7.2, 2 mmol L-glutamine, 5% fetal calf serum, 10^4^ units/ml penicillin, 1,250 units/ml Nystatin, 10 mg/ml streptomycin (Biological Industries, Beit Haemek), 5% tryptose phosphate broth (Gibco) in 35-mm diameter dishes were transiently transfected with > 75% efficiency with a mixture of 1.5 μg each of pcDNA-3′HIV plasmid and pcDNA3 control DNA or pEF5-E3L, vector expressing *VP35* [27], or vector expressing *PKR*, *eIF2*α*S51A*, *eIF2*α*S51D*, or *eIF2*α*wt*. The DNA was mixed with 6 μl Turbofect (Fermentas) and 100 μl 150 mmol NaCl for 10 min before addition to the cell culture. After 5 h, the medium was replaced by fresh culture medium.

### Northern blot analysis

Cells were washed with phosphate buffered saline, briefly incubated with 0.5 ml 0.05% trypsin-EDTA (Invitrogen) until they detached from the plate and resuspended in 1 ml 10% fetal bovine serum-containing medium to inactivate trypsin. Cells were pelleted at 2750 × g for 5 min, washed in 1 ml phosphate buffered saline, centrifuged at 2750 × g for 5 min, resuspended in 0.6 ml RLT buffer (QIAGEN) and homogenized with a QIAshredder column (QIAGEN). Total RNA was isolated with the RNeasy kit (QIAGEN), and contaminating DNA removed with RNase-free DNase (QIAGEN) added during the isolation procedure. Gel electrophoresis of RNA was performed at 100 V in a 1% agarose gel in 3-(N-morpholino)-propanesulfonic acid (MOPS) buffer (40 mmol MOPS, 10 mmol sodium acetate, pH 7.0) with 7% formaldehyde. To examine equal sample loading, the gel was stained with 2 µg/ml ethidium bromide for 20 min. Ribosomal RNA bands (18S and 28S) were visualized with UV light. The RNA was transferred overnight onto a positively charged nylon membrane (Boehringer Mannheim) by means of capillary force and crosslinked to the membrane using UV (254 nm, 0.12 J). The 373-bp EcoRV-HindIII fragment of HIV-rtTA encoding the U3/R region was ^32^P-labeled using High Prime DNA Labeling kit (Roche Diagnostics) and used as HIV-specific probe. Prehybridization and hybridization was done in ULTRAhyb buffer (Ambion) at 55 °C for 1 and 16 h, respectively. The membrane was then washed twice at room temperature for 5 min in low-stringency buffer (2 × saline-sodium citrate (SSC), 0.2% sodium dodecyl sulfate (SDS)) and twice for 10 min at 50 °C in high-stringency buffer (0.1 × SSC, 0.2% SDS). Images were obtained using the PhosphorImager (Amersham Biosciences) and band density quantified with ImageQuant software.

### Splicing assay by RNase protection analysis

Total RNA was isolated with TRIZOL Reagent (Invitrogen) and after DNase I digestion (Promega) was subjected to RNase protection analysis with RNases A and T1, using a genomic riboprobe to analyze splicing of HIV *rev/tat* intron. PCR on pcDNA-3’HIV DNA, using forward primer 5′-GGATGGAGTGGGACAGAG and reverse primer 5′-GCACAGGCTCCGCAGATCG, generated a DNA fragment that was inserted into pGEM-T (Promega) and after SacII digestion, used for riboprobe transcription. In the ^32^P-labeled antisense RNA probe (SP6 transcript), unspliced pre-mRNA protects 413 nt from cleavage and spliced mRNA protects 140 nt. Protected RNA fragments were separated by electrophoresis in 4% polyacrylamide/8 M urea gels. Band intensity in autoradiograms was quantitated using TotalLab software.

### Splicing assay by qRT-PCR

Total cellular RNA was harvested using TRIZOL Reagent (Invitrogen) and after DNase I digestion (Promega), cDNA synthesized in a 20-μl reaction volume from 500 ng RNA using Verso RT-PCR Kit containing RT Enhancer to remove contaminating DNA (ABgene). Quantitative real-time PCR was performed in triplicate using 5 μl diluted cDNA, 1 μl primers/probe mix with Taqman detection chemistry and 10 μl 2 × ABsolute Blue QPCR Mix (ABgene). Reactions were performed for 40 cycles (95 °C for 15 s, 60 °C for 30 s, and 72 °C for 15 s) using a Rotor-Gene 6000 instrument (Corbett Life Science). A standard curve was generated in each run to quantify the amount of amplicons. Amounts of unspliced precursor RNA and spliced mRNA were normalized to *EGFP* mRNA for transfection efficiency and to *β-actin* mRNA as internal normalizing standard [10]. Amplification efficiency of spliced and unspliced forms, determined by CT slope, was similar and at least 99%.

### *Rev/tat* qRT-PCR primers and probes

Spliced and unspliced *rev/tat* transcripts were determined by qRT-PCR, using primers and probes from PrimerDesign, UK specific for spliced RNA (primers: 5′-GCAGGAAGAAGCGGAGACA, 5′-TTGGGAGGTGGGTTGCTTT; probe: 5′-CTCCTCAAGGCAGTCAGACTCATCAAGTT) and unspliced RNA (primers: 5′-AGACTAATAGAAAGAGCAGAAGACA, 5′-GTAGCACTACAGATCATCAATATCC; probe: 5′-TGCCCCATTTCCACCCCCATCTCCA).

### Activation of recombinant PKR

To express rPKR in a form that can be activated by RNA, human *PKR* cDNA was inserted into pHTT7K [28] between the NdeI and PstI sites and λ protein phosphatase cDNA (New England Biolabs) at the PstI site; PKR was expressed in *Escherichia coli* Rosetta(DE3)pLysS (Invitrogen) as full-length N-terminally hexahistidine-tagged protein*.* rPKR was recovered from inclusion bodies by solubilization in 6 M urea buffer and loaded onto a His∙Bind column (Novagen) that was eluted stepwise with imidazole. rPKR recovered after dialysis was > 98% pure on SDS-12% PAGE and was stored in 0.05 M Tris-HCl pH 7.4, 0.05 M KCl, 5 mmol DTT and 20% glycerol.

DNA templates encoding the 3′-terminal TAR element and upstream pseudoknot under the T7 promoter were generated from wild-type, TAR*3R* or *P1b* mutant pcDNA-3′HIV DNA using forward primer 5′-TGTAATACGACTCACTATAGGACTGGGGAGTGGCGAGC and reverse primer 5′-GCAAGCTTTATTGAGGCTTAAG. For activation of PKR, uncapped 123-nucleotide transcripts were generated using T7 transcription kit (Promega) and purified twice by Sephadex G-50 gel chromatography followed by chromatography on CF-11 cellulose, washing with ethanol and eluting with water as described [29].

Activation of rPKR by RNA was determined in the presence of [*γ*-^32^P]ATP in 50 mmol Tris–HCl pH 7.5, 50 mmol KCl, 2 mmol MgCl_2_, and 0.02 mmol unlabeled ATP; mixtures (10 μl) were incubated for 20 min at 25** °C** and resolved by SDS-10% PAGE. A master mix of rPKR and labeled ATP was distributed to all reaction vessels and only the RNA was added individually. Hence, identical amounts of rPKR substrate were present in the different samples.

### Computational analysis of RNA structure

The HIV-1 and SIVcpz TAR motifs and surrounding sequences were analyzed for RNA secondary structure, including pseudoknots, using the PknotsRG tool (https://bibiserv2.cebitec.uni-bielefeld.de). The pre-made alignment of all complete sequences for HIV-1 group M isolates (2017; without recombinants) from the Los Alamos HIV databases (www.hiv.lanl.gov) was used to analyze conservation of the RNA structure.

### Statistical analysis

Splicing efficiency and activation of recombinant PKR were compared using the one-tailed unpaired Student's *t*-test; *p < 0.05, **p < 0.005, ***p < 0.001; ns, not significant.

## Results

### Inhibitors of PKR activation repress HIV mRNA expression

**Fig. 1:**
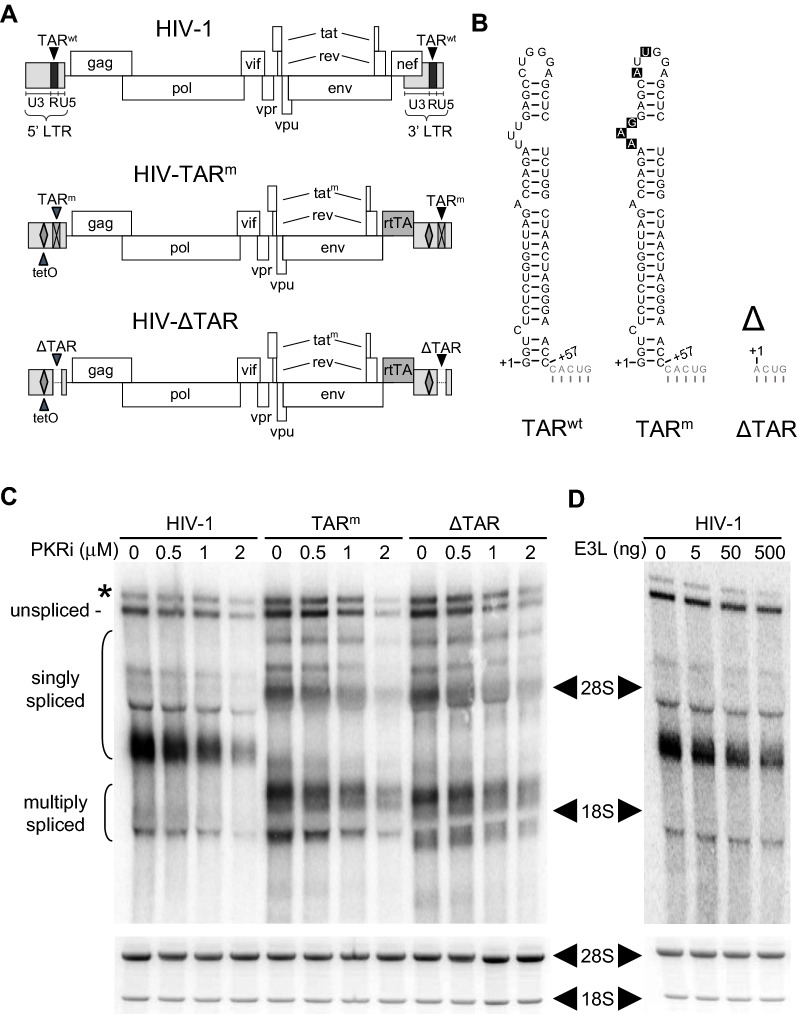
Production of HIV-1 mRNA species is sensitive to PKR antagonists. **A** Proviral DNA genome maps of HIV-1 LAI (TAR^wt^), HIV-rtTA (TAR^m^) and HIV-rtTA-ΔTAR (ΔTAR), with the long terminal repeat (LTR) subdivided into U3, R, and U5 domains. **B** TAR secondary structures followed by the first five nucleotides of the adjacent poly(A) hairpin (light gray). The Tat/TAR axis of transcriptional regulation was inactivated in TAR^m^ by nucleotide substitutions in the bulge and loop. The HIV-rtTA variant lacking TAR (ΔTAR) has a new transcription start site at the second nucleotide of the poly(A) hairpin [22]. **C** Human HEK-293 T cells were transfected with a vector carrying the HIV-1, TAR^m^ or ΔTAR genome, in the absence or presence of the indicated concentrations of PKR inhibitor (PKRi). Total RNA was isolated 48 h after transfection and analyzed by northern blot using a probe that detects all HIV-1 RNA variants. Unspliced (9 kb), singly spliced (4 kb) and multiply spliced (2 kb) RNA size classes [30] and a band resulting from transcriptional read-through on the vector (*)[57] are indicated. The transcripts observed for the different virus constructs vary in size due to the mutations introduced to generate the HIV-rtTA variants, TAR^m^ and ΔTAR, including deletions and insertions (rtTA replaced Nef, different 3’UTR). Bottom panel shows 18S and 28S ribosomal RNA loading controls (ethidium bromide staining). A representative of 3 experiments is shown. **D** HEK-293 T cells were transfected with vector carrying the HIV-1 genome, in the absence or presence of the indicated amounts of Vaccinia E3L expression vector (E3L, ng DNA per transfection). Total RNA was isolated and analyzed by Northern blotting as in **C**. A representative of 3 experiments is shown

TNF-*α*, a cytokine pivotal for protective immunity, is expressed promptly during inflammatory responses; in human peripheral blood mononuclear cells, *TNF-α* mRNA becomes maximally expressed within 3 h [12]. Efficient *TNF-*α mRNA splicing is achieved through a 104-nucleotide RNA element in the 3’-UTR that activates PKR [13]. The *TNF-α* RNA activator of PKR causes mRNA splicing to be sensitive to 2-aminopurine, an eIF2α kinase inhibitor [12, 13]. The RNA element in *TNF-*α pre-mRNA that activates PKR renders splicing not only fully dependent on PKR activation but also highly efficient [10, 13]. The 59-nucleotide TAR stem-loop element that is present at the 5′ and 3′ termini of HIV transcripts (Fig. 1A, B) activates purified recombinant human PKR in vitro [18]. Potentially, therefore, TAR might, as for *TNF-α*, serve in the function of PKR activator to render splicing of HIV mRNA efficient.

HIV expresses a full length ~ 9 kb transcript, used as mRNA for Gag and Pol production and as viral genome, and a large variety of singly spliced (~ 4 kb) and multiply spliced (~ 2 kb) transcripts for all other viral proteins, including Rev [30, 31]. To examine whether the production of HIV mRNA might depend on PKR, we transfected human HEK-293 T cells with a plasmid carrying the complete wild type HIV-1 genome (LAI strain) and quantitated the expression of various HIV mRNA species in the presence of increasing doses of a small-molecule inhibitor of the kinase catalytic site in PKR, PKRi. Northern blot analysis showed that PKRi progressively inhibited production of all HIV mRNA species, including singly spliced and multiply spliced mRNAs (Fig. 1C and Additional file 1: Fig. S1). A similar pattern of inhibition of mRNA species encoded by HIV was observed when instead of PKRi, we co-expressed the Vaccinia E3L protein, a viral PKR antagonist [23] that strongly attenuates the action of the intragenic *IFN-γ* RNA activator of PKR [9] (Fig. 1D). E3L competes with PKR in binding to the activating RNA, forming an E3L-PKR-RNA complex in which the *N*-terminal half of E3L interacts physically with the protein kinase domain of PKR [23]. The independent results with PKRi and E3L support a role for PKR activation in the control of HIV mRNA expression.

To study the possible role of TAR in PKR activation, we used an HIV genomic construct (TAR^m^) in which the Tat-TAR transcription mechanism is inactivated through mutations in Tat and TAR (nucleotide substitutions in the bulge and loop sequence) and functionally replaced by the integrated doxycycline-inducible Tet-On gene regulation system [21]. TAR^m^ does not depend on TAR for the activation of transcription, which makes it possible to study other functions of TAR in gene expression. As for the wild type virus, expression of the various mRNA classes by TAR^m^ was sensitive to PKRi (Fig. 1C and Additional file 1: Fig. S1). We also tested RNA production of a TAR^m^ derivative that lacks both 5’ and 3’ TAR elements and, like TAR^m^, replicates efficiently in infected cell cultures [22]. This ∆TAR variant produced similar amounts of the different viral RNA classes as TAR^m^ and their production was inhibited likewise by PKRi (Fig. 1A, C and Additional file 1: Fig. S1). These results suggest that the HIV RNA genome may harbor, in addition to the TAR element, another activator of PKR.

### Splicing of *rev/tat* mRNA is regulated by activation of PKR

The inhibition of the production of all size classes of HIV mRNA by PKRi and by E3L, including that of the unspliced RNA (Fig. 1C, D), did not resolve whether splicing or another expression step requires the activation of PKR. Splicing effects are difficult to interpret within the complete HIV genome context. For example, decreased splicing will lower the production of the regulatory Tat and Rev proteins, both encoded by multiply spliced mRNAs [32]. Tat activates viral transcription and influences splicing of the viral RNAs [33, 34], whereas Rev stimulates nuclear export of unspliced and singly spliced RNAs, which reduces splicing [35, 36]. Unspliced RNA precursors that are retained in the nucleus may be degraded.

**Fig. 2:**
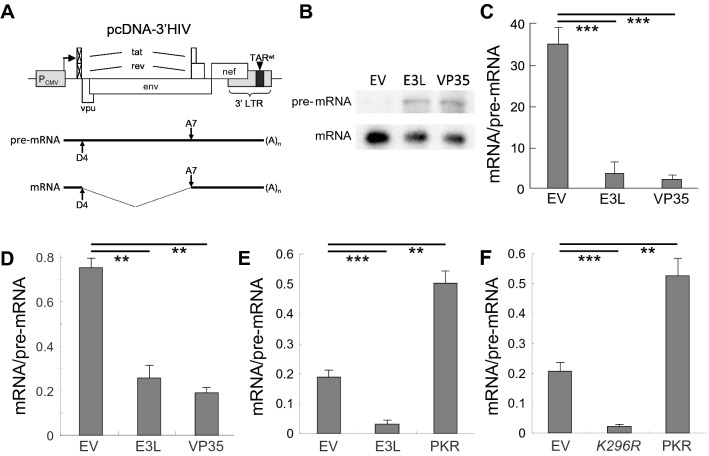
Expression of viral PKR antagonist protein Vaccinia E3L or Ebola VP35 inhibits excision of HIV *rev/tat* intron. **A** Vector encoding the 3’ half of the HIV genome. Vector pcDNA-3’HIV carries the 3’ portion of the HIV-1 genome expressed under the constitutive cytomegalovirus (CMV) promoter. The viral sequences start downstream of the *rev* AUG translation start codon to prevent Tat and Rev production. The construct retains splice donor D4 (5’ss #4) and splice acceptor A7 (3’ss #7), allowing for a single splicing event of the *rev/tat* intron (bottom). **B, C** E3L and VP35 inhibit splicing of *rev/tat* mRNA. BHK-21 cells were cotransfected with 1 µg of pcDNA-3’HIV DNA together with 1 µg pBS empty vector (EV), E3L expression vector (E3L) or VP35 expression vector (VP35). Total RNA was isolated at 18 h post-transfection. Unspliced pre-mRNA (413 nt) and spliced *rev/tat* mRNA (140 nt) were determined by RNase protection analysis (**B**). The top autoradiogram (pre-mRNA) underwent a longer exposure. Band intensity was quantitated and the ratio of spliced over unspliced RNA within each lane, which reflects splicing efficiency, is plotted in bar graph (**C**) (error bars, SEM; n = 3). A representative experiment is shown. **D** E3L and VP35 inhibit splicing of *rev/tat* mRNA. In independent transfections, performed as in **B**, total RNA was isolated at 12 h; spliced and unspliced *rev/tat* transcripts were determined by qRT-PCR. Splicing efficiency is expressed as mRNA/pre-mRNA ratio (error bars, SEM; n = 3). **E** Whereas *rev/tat* intron excision is inhibited by co-expression of E3L, it is stimulated by co-expression of PKR. BHK-21 cells were cotransfected with 1 µg of pcDNA-3’HIV DNA together with 1 µg pBS empty vector (EV) or with vector expressing E3L or human PKR. Total RNA was isolated at 12 h post-transfection. Spliced and unspliced *rev/tat* transcripts were determined by qRT-PCR. Splicing efficiency is expressed as mRNA/pre-mRNA ratio (error bars, SEM; n = 3). **F** Splicing of *rev/tat* mRNA is blocked by expression of *K296R trans*-dominant negative mutant PKR*.* BHK-21 cells were cotransfected with 1 µg of pcDNA-3’HIV DNA together with 1 µg pBS empty vector (EV) or with vector expressing mutant *K296R* or human PKR. Total RNA was isolated at 12 h post-transfection. Spliced and unspliced *rev/tat* transcripts were determined by qRT-PCR. Splicing efficiency is expressed as mRNA/pre-mRNA ratio (error bars, SEM; n = 3)

Given the complexity of full-length HIV expression and its multiple splicing products, we created a vector expressing the 3’ half of the HIV genome, including the large *rev/tat* intron whose excision constitutes the sole splicing event for RNA encoded by this vector (pcDNA-3′HIV) (Fig. 2A). Compared to full-length HIV (Fig. 1A), the 3′HIV construct covers 40% of the nucleotide sequence, including the complete 3′ domain (Fig. 2A). To avoid confounding effects on splicing by the Tat and Rev proteins, the viral sequences in this vector start downstream of the *rev* and *tat* AUG translation start codons, so that no functional Tat and Rev can be produced. Moreover, the construct lacks the 5’ TAR element that enhances splicing at the major splice donor site via the Tat protein [33, 34]. Upon transfection of this vector into cells, we monitored splicing of the pre-mRNA transcript containing the *rev/tat* intron by quantitating unspliced and spliced RNA. Ribonuclease protection analysis (Fig. 2B, C; the ratio of spliced over unspliced RNA within each individual lane in Fig. 2B reflects splicing efficiency plotted in Fig. 2C) and quantitative real-time polymerase chain reaction (qRT-PCR) analysis (Fig. 2D) each showed that the transcript was spliced efficiently, resulting in a high mRNA/pre-mRNA ratio.

Excision of the *rev/tat* intron was inhibited strongly by co-expression of the viral PKR antagonist proteins, Vaccinia E3L and Ebola VP35 [27, 37], resulting in accumulation of the unspliced pre-mRNA and reducing the mRNA/pre-mRNA ratio which denotes splicing efficiency (Fig. 2B–D). By contrast, overexpression of PKR in the cell enhanced *rev/tat* intron excision, reflected by a significant increase in mRNA/pre-mRNA ratio (Fig. 2E and F). Notably, as shown in Fig. 2F, splicing of *rev/tat* mRNA was abrogated by expression of a *trans*-dominant negative mutant of PKR, K296R, that blocks the activation of PKR in the cell [23].

These results demonstrate a positive and indispensable role for PKR activation in HIV *rev/tat* mRNA splicing.

### Phosphorylation of eIF2α controls *rev/tat* mRNA splicing

**Fig. 3:**
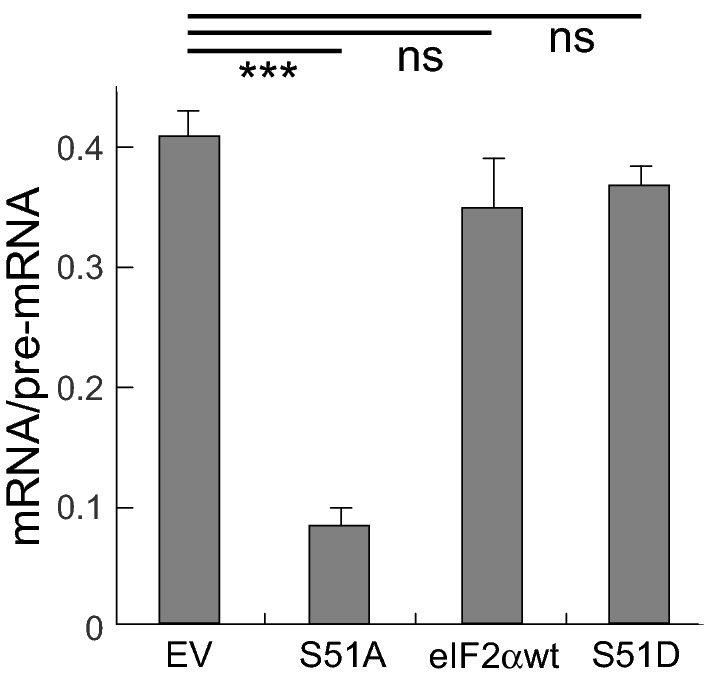
PKR-dependent *rev/tat* mRNA splicing requires eIF2α phosphorylation. Expression of non-phosphorylatable eIF2αS51A, yet not of phosphomimetic eIF2αS51D, inhibits splicing. BHK-21 cells were cotransfected with 1.5 µg of pcDNA-3’HIV DNA together with 1.5 µg DNA of pBS empty vector (EV), eIF2αS51A expression vector (S51A), vector expressing wild type eIF2α (eIF2αwt) or vector expressing eIF2αS51D (S51D). Total RNA was isolated at 20 h post-transfection. Spliced and unspliced *rev/tat* transcripts were determined by qRT-PCR. Splicing efficiency is expressed as mRNA/pre-mRNA ratio (error bars, SEM; n = 3). A representative experiment is shown

Repression of mRNA translation by PKR depends strictly on phosphorylation of the translation initiation factor protein chain, eIF2α, at its sole phosphorylation site, Serine51 [3, 38]. Expression of non-phosphorylatable mutant eIF2α, eIF2αS51A, but not of wild type eIF2α, abrogates efficient splicing of *TNF-*α [10] and *globin* pre-mRNA [14], showing that splicing driven by the *TNF-α* and *globin* RNA activators of PKR depends tightly on eIF2α phosphorylation*.* eIF2*α*S51A inhibits eIF2α phosphorylation by activated PKR [10]. Indeed, expression of non-phosphorylatable mutant eIF2αS51A, but not of wild type eIF2α, abrogated efficient splicing of *rev/tat* mRNA encoded in pcDNA-3’HIV (Fig. 3). qRT-PCR analysis shows that pre-mRNA was converted effectively into mRNA when empty control vector or wild type eIF2α vector was expressed, whereas co-expression of eIF2αS51A led to a strong decline in splicing efficiency. By contrast, as for splicing of *TNF-*α [10] and *globin* pre-mRNA [14], expression of eIF2αS51D, a phosphomimetic mutant of eIF2α that inhibits translation [39], did not significantly affect *rev/tat* mRNA splicing, indicating a requirement for authentic phosphorylated eIF2α (Fig. 3). Unlike eIF2αS51A, eIF2αS51D does not inhibit eIF2α phosphorylation by activated PKR [10].

These results reveal an essential function for eIF2α phosphorylation in HIV *rev/tat* mRNA splicing that accounts for the need for PKR activation.

### Nature of the RNA activator of PKR in HIV pre-mRNA

**Fig. 4:**
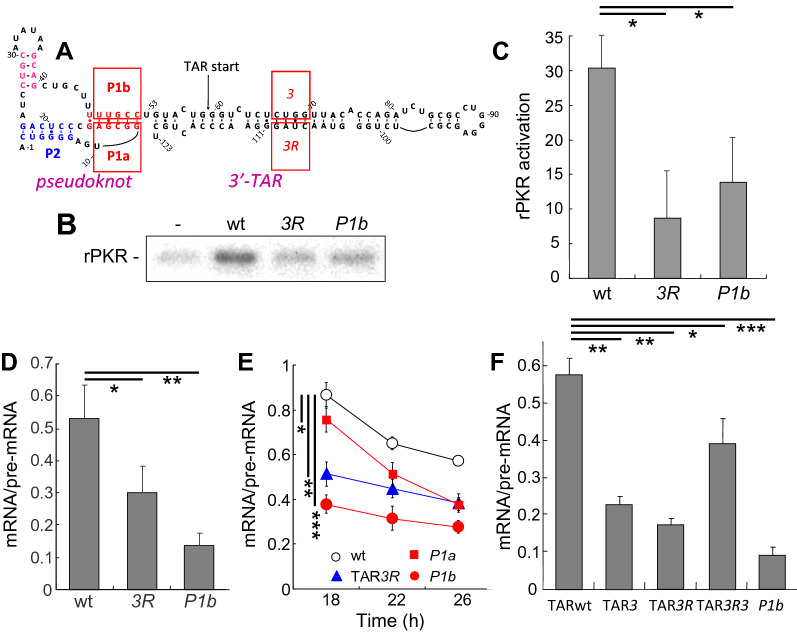
3’-Proximal RNA pseudoknot is essential for activation of PKR and splicing. **A** Secondary structure of the 123-nucleotide region in the HIV-1 3’-UTR that contains the 3’-terminal TAR element and upstream pseudoknot. Pseudoknot stems P1 and P2 are indicated. Boxed nucleotides in P1 were mutated to the nucleotide sequence in the complementary strand (*P1b*, UUGCC > AGCGG; *P1a*, AGCGG > UUGCC). The *3R* mutation in TAR was CUAG > UGGC; the *3* mutation in TAR was CUGG > GCCA [18]. **B, C** Both intact 3′-terminal TAR and pseudoknot stem P1 are required for PKR activation. Activation of PKR was assayed using rPKR (85 ng per lane) in the absence of RNA (-) or in the presence of wild type (wt), TAR*3R* or *P1b* mutant transcript at 0.1 μg/ml RNA. Position of phosphorylated rPKR (68 kDa) is indicated. A representative experiment is shown (**B**). Band intensity was quantitated and is plotted in bar graph (**C**), subtracting the value in the absence of RNA (error bars, SEM; n = 3). **D** Mutation of TAR or pseudoknot stem P1 impairs *rev/tat* splicing efficiency. BHK-21 cells were transfected with 3 µg of pcDNA-3’HIV DNA wt, TAR*3R* or *P1b*. Total RNA was isolated at 20 h and spliced and unspliced *rev/tat* transcripts were determined by qRT-PCR. Splicing efficiency, expressed as mRNA/pre-mRNA ratio, was determined for each DNA construct and corrected for between-session variation [58] (error bars, SD). **E** Mutation of each strand within pseudoknot stem P1 affects *rev/tat* splicing efficiency. BHK-21 cells were transfected with 3 µg of pcDNA-3’HIV DNA wild type (wt) or mutant forms *P1b*, *P1a* or TAR*3R.* Total RNA was isolated at the indicated times post-transfection. Spliced and unspliced *rev/tat* transcripts were determined by qRT-PCR. Splicing efficiency is expressed as mRNA/pre-mRNA ratio (error bars, SEM; n = 3). A representative experiment is shown. **F** Reduction in *rev/tat* intron splicing within the cell by TAR*3* and TAR*3R* mutations and partial restoration by double mutation TAR*3R3*. BHK-21 cells were transfected with 3 µg of pcDNA-3’HIV DNA wild type (wt) or mutant forms *P1b*, TAR*3*, TAR*3R* or TAR*3R3.* Total RNA was isolated at 18 h post-transfection. Spliced and unspliced *rev/tat* transcripts were determined by qRT-PCR. Splicing efficiency is expressed as mRNA/pre-mRNA ratio (error bars, SEM; n = 3)

The 59-nucleotide TAR stem-loop is present at the 3′ end of the transcript encoded by the pcDNA-3’HIV vector (Fig. 2A). The 123-nucleotide 3’-terminal fragment of *rev/tat* pre-mRNA, including TAR (Fig. 4A), can activate recombinant human holo-PKR in vitro [18] and this activation was impaired severely by the TAR*3R* mutation [18] that replaces 4 nucleotides in the TAR stem, thereby abolishing base-pairing (Fig. 4A, B). Quantitation of phosphorylated recombinant PKR band intensity in Fig. 4B is presented in Fig. 4C. To examine whether TAR supports *rev/tat* mRNA splicing, we introduced the TAR*3R* mutation into pcDNA-3’HIV and analyzed the production of spliced and unspliced transcripts in transfected cells. Indeed, qRT-PCR analysis showed that the TAR*3R* mutation causes a significant reduction in *rev/tat* intron excision (Fig. 4D).

As noted above, the finding that production of mRNA by TAR-deficient HIV ∆TAR is sensitive to PKRi (Fig. 1C and Additional file 1: Fig. S1), supports the concept that the HIV genome contains an additional activator of PKR. As shown by mutational analysis, the activators of PKR in *TNF-α* pre-mRNA and *IFN-γ* mRNA fold into compact RNA pseudoknots [9–11]. Using bioinformatic analysis, we observed that the sequence just upstream of TAR in the 3′-terminal region of HIV-1 mRNA has the potential to fold into an RNA pseudoknot (Fig. 4A). This small pseudoknot had not been detected by chemical probing analysis of full-length HIV-1 RNA [40]. Yet, within the sequence just upstream of TAR, chemical probing supports two pseudoknot stems having moderate stability, consistent with dynamic refolding of the compact pseudoknot (Additional file 1: Fig. S2). Within the cell, HIV-1 RNA is highly dynamic in its folding, evident from the widespread heterogeneous nature of HIV-1 RNA structure conformation [41]. Notably, the short AGU linker connecting pseudoknot stems P1 and P2 is unreactive to chemical probing (Additional file 1: Fig. S2), indicative of structural constraint that may reflect the properties of the compactly folded *TNF-*α pseudoknot that functions as activator of PKR in splicing [10].

To evaluate whether the HIV pseudoknot might function as an activator of PKR in splicing, we examined PKR activation by a mutant 123-nucleotide 3’-terminal transcript in which formation of the putative pseudoknot RNA helix P1 is abrogated by base substitutions in the following strand (*P1b*) that replaces 5 nucleotides by the nucleotide sequence in the complementary strand, thereby abolishing base-pairing (Fig. 4A). As seen in Fig. 4B and C, not only TAR*3R* but also the *P1b* mutation impaired the ability to activate recombinant human PKR. PKR was phosphorylated far less when the RNA was mutated, reflecting strongly reduced PKR activation (Fig. 4B, C). Moreover, introduction of the *P1b* mutation into pcDNA-3’HIV strongly reduced the splicing efficiency of HIV *rev/tat* intron, even exceeding the reduction in splicing by the TAR*3R* mutation (Fig. 4D, E). The clear splicing phenotype of mutant *P1b* motivated mutation of the leading strand in helix P1, to yield mutant *P1a* that also replaces 5 nucleotides by the nucleotide sequence in the complementary strand, to abolish base-pairing (Fig. 4A). The *P1a* mutation likewise impaired splicing efficiency of *rev/tat* mRNA, though less severely than did *P1b* (Fig. 4E). These mutations validate the positive role of the pseudoknot in promoting splicing.

In view of the pronounced splicing phenotypes of mutants *P1b* and *P1a* (Fig. 4D, E), we created double mutant *P1ab* in which 5 base pairs in helix P1 are restored, albeit in the inverse orientation. In this regard, it should be noted that the pseudoknotted RNA activators of PKR in human *IFN-γ* mRNA [11] and *TNF-α* pre-mRNA [10] each lost the ability to activate PKR when only a single base pair was inverted within their RNA structure. However, upon transfection of the *P1ab* double mutant vector, in which 5 base pairs were inverted, no expression of pre-mRNA or mRNA could be detected using qRT-PCR (data not shown), documenting the sensitivity of this stem and precluding further analysis.

The extensively linear double-stranded RNA motif within the TAR element (Fig. 4A) allows the TAR*3R* mutation to be compensated by the complementary TAR*3* mutation for activating PKR in vitro [18]. Indeed, although the TAR*3R* and TAR*3* mutations each diminished *rev/tat* intron excision, albeit less severely than did pseudoknot mutation *P1b*, splicing within the cell could be restored in part by the double mutation TAR*3R3* (Fig. 4F). These results demonstrate a collaborative role for the pseudoknot and the 3’-terminal TAR stem-loop in mediating PKR-regulated splicing of the *rev/tat* intron, the pseudoknot being dominant (Fig. 4D–F).

**Fig. 5:**
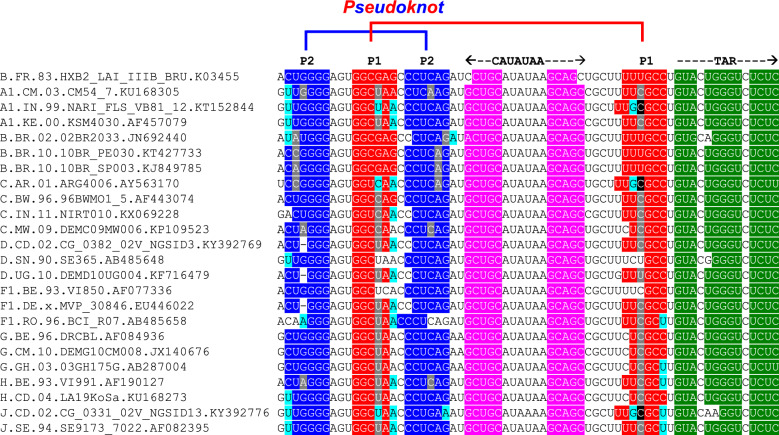
Conservation of HIV pseudoknot stems P1 and P2. Nucleotide sequence alignment of the pseudoknot and TAR start in LAI and other HIV-1 isolates. Colors mark base-paired nucleotides in pseudoknot stems P1 (red) and P2 (blue), in the stem of the TATA box loop (nucleotides 27–40 in Fig. 4A) (magenta) and in TAR (green). Grey boxes denote nucleotide differences that prevent base pairing; cyan boxes denote nucleotide differences that do not abolish base pairing. Black boxes denote bulged nucleotides. HIV-1 group M isolate sequences (region 9481–9547) corresponding to different subtypes were retrieved from http://www.hiv.lanl.gov/ (year 2017). Representative examples from a deeper conservation analysis are shown. For subtypes with more than 150 entries, the 1st, 50th, 100th and 150th isolates are presented (subtype B and C); for subtypes occurring less than 150 times, the 1st, 10th and 20th isolate are presented (A1, D, F1 and G); and for subtypes occurring less than 20 times, the 1st and 5th isolate are presented (H and J). In case of sequence uncertainty or incompleteness, the next complete isolate was selected

**Fig. 6:**
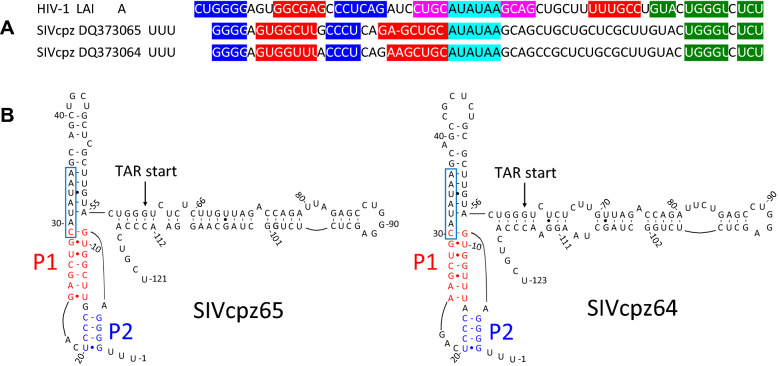
Pseudoknot stems P1 and P2 are conserved between HIV-1 and SIVcpz. **A** Alignment of the region upstream of 3’-TAR in the HIV-1 LAI strain (Genbank reference K03455) with simian immunodeficiency virus strains SIVcpzEK505 (DQ373065, SIVcpz65) and SIVcpzLB7 (DQ373064, SIVcpz64). Nucleotide coloring marks base pairs within pseudoknot stems P1 (red), P2 (blue), and TAR (dark green). Cyan color marks the TATA box sequence (CATATAA; [59]) that functions at the 5’terminus of HIV DNA as transcription promoter element. **B** Folding of the pseudoknot as predicted for the SIVcpz strains. SIVcpz RNA contains an additional helix between the pseudoknot and TAR wherein the TATA box is located (boxed). Representative examples from a deeper conservation analysis are shown

We next examined whether the two pseudoknot stems P1 and P2 are conserved among different HIV-1 strains and related simian immunodeficiency viruses (SIV). In both *TNF-*α pre-mRNA and *IFN-γ* mRNA, the pseudoknot elements show phylogenetic conservation [9–11]. Indeed, the potential to form the HIV pseudoknots is conserved broadly among isolates belonging to different HIV group M subtypes (Fig. 5). HIV-1 comprises three major groups: M, N, and O. Group M (Major) viruses causes more than 90% of all HIV/AIDS cases and is divided into subtypes A-K. Its zoonotic origin is SIV chimpanzee (SIVcpz). Group N stands for “Non-M and Non-O” and its occurrence is very rare. Until recently, only 20 group N cases have been recorded [42]. HIV-1 Group O (Outlier) and HIV-2 both originated from a zoonotic origin distinct from that of group M. The origin of group O is SIV gorilla (SIVgor), resulting from transmission of SIVcpz to gorilla and the source of HIV-2 was sooty mangabey (SIVsmm) [43]. SIV isolates show major differences from HIV in their TAR domains [44]. Notably, although the nucleotide sequence in the region preceding TAR differs markedly in closely related SIV chimpanzee (SIVcpz) isolates, it can fold into a similar pseudoknot structure by base pairing of different sequence segments, exemplified for two representative isolates (Fig. 6A, B).

The impairment of *rev/tat* pre-mRNA splicing mediated by a TAR mutation, together with the clear splicing-defective phenotypes of the pseudoknot mutations, demonstrate that the conserved pseudoknot and TAR each promote splicing independently by activating PKR and inducing eIF2α phosphorylation. Thus, unlike the case for *TNF-α* and *globin* pre-mRNA, the HIV-1 RNA activator of PKR that drives splicing of *rev/tat* mRNA is composite in nature. Conceivably, HIV evolved to harbor dual RNA activators of PKR to ensure efficient splicing.

## Discussion

**Fig. 7:**
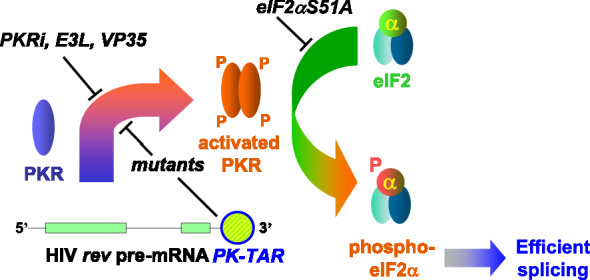
Activation of PKR by *rev/tat* pre-mRNA triggers splicing via eIF2α phosphorylation. Activation of PKR occurs through collaborative action of the 3′-terminal TAR element and the pseudoknot (PK) located just upstream in *rev/tat* pre-mRNA. The helical structures generated by the pseudoknot and by TAR facilitate PKR dimerization and activation. Once activated, PKR catalyzes phosphorylation of eIF2α, which mediates efficient splicing. Activation of PKR is inhibited by mutations in TAR or pseudoknot stem P1, as well as by PKRi and expression of E3L or VP35; phosphorylation of eIF2α by PKR is inhibited by expression of non-phosphorylatable mutant eIF2S51A. The need for PKR activation and eIF2α phosphorylation links HIV-1 mRNA splicing to the integrated stress response

The canonical function of the RNA-dependent stress response kinase PKR is to downregulate translation by phosphorylating the initiation factor eIF2α chain. Our finding is that this negative translational control mechanism, hitherto thought to be a cornerstone of antiviral defense, positively regulates HIV mRNA splicing. We show that splicing of the large *rev/tat* intron, located in the 3’-proximal region of the HIV genome, requires the activation of PKR and phosphorylation of eIF2α (Fig. 7). As such, HIV employs the same mechanism to upregulate its mRNA splicing that is used exceptionally by the human inflammatory cytokine *TNF-*α gene and the *globin* genes to render splicing of their pre-mRNAs highly efficient [10, 14].

Conceivably, HIV may have acquired PKR-dependent control of its *rev/tat* mRNA splicing in the course of its evolution, becoming integrated into the host genome where once entering active replication, its expression needed to become highly efficient as if it were a cellular gene essential for survival, as exemplified by the human *TNF-α* and *globin* genes.

Experiments monitoring expression of full-length HIV-1 revealed that different PKR inhibitors did not only cause a decrease of the multiply spliced *rev/tat* mRNAs (Fig. 1), but production of all HIV mRNA species, including unspliced and singly spliced ones, was reduced in a dose-dependent manner by Vaccinia E3L and by a small-molecule inhibitor of PKR. However, as pointed out above (Section: Splicing of *rev/tat* mRNA is regulated by activation of PKR), splicing effects are difficult to interpret within the complete HIV genome context, as a decrease in splicing will also decrease the production of Tat and Rev proteins that will, in turn, affect RNA production and processing, masking thereby a specific effect on splicing. To deconvolute the molecular mechanism that controls PKR-dependent splicing of HIV-1, we created a vector harboring 40% of the HIV nucleotide sequence, including its complete 3’ domain, in which excision of the large *rev/tat* intron could be studied without confounding effects of Tat and Rev proteins. Co-expression of proteins used by viruses to inhibit the activation of PKR in order to evade the interferon-induced antiviral response, Vaccinia E3L and Ebola VP35, led to severe inhibition of HIV *rev/tat* intron excision, as did co-expression of a *trans*-dominant negative mutant of PKR that blocks phosphorylation of the kinase essential for its activation, whereas overexpression of PKR stimulated splicing significantly. Consistent with the need for PKR activation in HIV *rev/tat* mRNA splicing, a portion of PKR resides in the nucleoplasm in an underphosphorylated state [45]. Co-expression of a non-phosphorylatable mutant of eIF2α, eIF2αS51A, strongly inhibited splicing whereas expression of a phosphomimetic mutant of eIF2α, eIF2αS51D, failed to stimulate *rev/tat* mRNA splicing, even though eIF2αS51D mimics phosphorylated eIF2α in inhibiting translation [39]. This supports the conclusion that, as for the *TNF-*α gene and the *globin* genes [10, 14], authentic phosphorylated eIF2α is needed to render *rev/tat* mRNA splicing efficient.

The exceptional stability of *β-globin* pre-mRNA permitted the analysis of PKR-dependent splicing in nuclear extract of human cells, including interaction of this pre-mRNA with the splicing machinery during spliceosome formation [14]. Early spliceosome assembly on *β-globin* pre-mRNA, formation of complex A, depends strictly on the activation of PKR and on phosphorylated eIF2α [14]; hence, this requirement plausibly underlies the present observations on splicing of *rev/tat* mRNA.

The BHK-21 and human HEK-293 T cell lines used in this study were used previously to deconvolute the mechanisms of PKR-dependent regulation of the human *IFN-γ* and *TNF-α* genes, as well as of the α*-globin* and *β-globin* genes [9, 10, 14]. Using similar experimental designs, these cells allowed for in-depth analysis of the molecular mechanism underlying PKR-dependent control of HIV *rev/tat* splicing, revealing a mechanism that had eluded research on HIV-1 biology during several decades. Even though the control of *globin* gene expression has long served as a paradigm in molecular biology, including in the demonstration of splicing of cellular mRNA [46, 47], the PKR dependence of this process was revealed only recently [14]. In the case of *TNF-*α, it was possible to validate that the dependence of pre-mRNA splicing on eIF2α phosphorylation in BHK cells operates in human peripheral blood mononuclear cells that are the natural cells expressing this gene [10]. Moreover, mutations that affect base pairing within a critical helix of the RNA activator of PKR in *β-globin* pre-mRNA and thereby impair splicing in HEK-293 T cells [14] are associated with human *β*-thalassemia [48]. It should be noted that several breakthrough findings on HIV-1 gene regulation are based on experiments where a vector expressing part of the HIV-1 genome in cell lines was used to deconvolute molecular mechanisms that control HIV-1 gene expression [49–52]. These findings strengthen the concept that the present results have direct relevance to HIV-1 biology.

We describe a highly conserved RNA pseudoknot structure, located just upstream of the 3’-terminal TAR element in *rev/tat* pre-mRNA. Mutations within this pseudoknot impaired splicing efficiency, showing its essential role in HIV *rev/tat* mRNA splicing. Such a mutation impaired the ability of the 3’-terminal fragment of HIV RNA to activate PKR. The finding that the potential of the viral RNA to fold into this pseudoknot is conserved across a broad range of HIV isolates supports the concept that its function in virus replication is essential. Within the *IFN-γ* RNA pseudoknot that activates PKR, the kinase protects at least one of the nucleotides in each of four orientation-sensitive base pairs from cleavage [11]; likewise, PKR may well stabilize the HIV pseudoknot through RNA–protein contacts. In this context, the *3* and *3R* mutations in the RNA stem of TAR, which impair the ability of TAR RNA to activate PKR in vitro [18], each also impaired excision of *rev/tat* intron. The results of Fig. 4 show that the TAR element activates PKR within the cell and thereby impacts positively on splicing of the viral RNA.

Based on how mutation of these two distinct RNA elements impacts on their function in activating PKR and in enabling splicing (Fig. 4), we demonstrated here a collaborative role for the pseudoknot and TAR in inducing PKR-regulated splicing of the *rev/tat* intron. Splicing at other 5’ and 3’ splice sites may depend similarly on these elements. RNA secondary structures reported previously to impact HIV splicing do not act by activating PKR. Earlier work on HIV splicing dealt with RNA stem-loop structures, such as the major 5’ splice donor site, that affect splice site efficiency by recruitment of splicing regulatory proteins, an altogether different subject [53–55]. Multiple levels of inhibition by PKR during HIV replication were described [56].

Interaction of eIF2 with the 5’-terminal TAR loop structure allows HIV-1 mRNA to compete more effectively during protein synthesis [19], indicating that the association of eIF2 with 5’-terminal TAR does not reinforce local phosphorylation of eIF2α by PKR.

## Conclusions

The present results on HIV provide the first example of a virus co-opting activation of PKR by its RNA, a cellular antiviral mechanism, to promote splicing. In this respect, HIV subverts the strategy of the cellular pro-inflammatory *TNF-α* gene for its own benefit. Our findings raise the question whether other viruses may use local activation of PKR through RNA elements within their genome to achieve efficient splicing of their mRNA.

## Supplementary Information

**Additional file 1: Fig. S1.** PKR inhibitor impairs expression of all size classes of HIV mRNA. A Representative mRNA species from each mRNA class in Fig. 1C (unspliced, 9 Kb; singly spliced, 4 Kb; multiply spliced, 2 Kb) are denoted by red asterisks. The shift in spliced mRNA banding pattern between the HIV-1 wt and the HIV-rtTA variants (TARm and ΔTAR) is caused by sequence differences (rtTA replaced Nef, different 3’UTR). B The denoted species from each mRNA class were quantified using ImageJ software (https://imagej.nih.gov/) (PKRi, μM). **Fig. S2.** Chemical reactivity of HIV pseudoknot stems P1 and P2. Secondary structure upstream of TAR and lower part of the TAR stem are shown for NL4-3 HIV-1 RNA. G labeled (*) is C in HIV-1 LAI. Chemical reactivity by SHAPE is shown, colors denote reactivity as high (red), moderate (orange), low (green) and little or none (black) [40].

## Data Availability

Source data are provided with this paper. All other datasets generated and analyzed in the current study are available from the corresponding author upon reasonable request.

## References

[1] Harding HP, Zhang Y, Zeng H, Novoa I, Lu PD, Calfon M, Sadri N, Yun C, Popko B, Paules R, Stojdl DF, Bell JC, Hettmann T, Leiden JM, Ron D (2003). An integrated stress response regulates amino acid metabolism and resistance to oxidative stress. Mol Cell.

[2] Muaddi H, Majumder M, Peidis P, Papadakis AI, Holcik M, Scheuner D, Kaufman RJ, Hatzoglou M, Koromilas AE (2010). Phosphorylation of eIF2α at serine 51 is an important determinant of cell survival and adaptation to glucose deficiency. Mol Biol Cell.

[3] Sonenberg N, Hinnebusch AG (2009). Regulation of translation initiation in eukaryotes: mechanisms and biological targets. Cell.

[4] Stark GR, Kerr IM, Williams BR, Silverman RH, Schreiber RD (1998). How cells respond to interferons. Annu Rev Biochem.

[5] Kaufman RJ (1999). Double-stranded RNA-activated protein kinase mediates virus-induced apoptosis: a new role for an old actor. Proc Natl Acad Sci USA.

[6] Zhang F, Romano PR, Nagamura-Inoue T, Tian B, Dever TE, Mathews MB, Ozato K, Hinnebusch AG (2001). Binding of double-stranded RNA to protein kinase PKR is required for dimerization and promotes critical autophosphorylation events in the activation loop. J Biol Chem.

[7] Dey M, Cao C, Dar AC, Tamura T, Ozato K, Sicheri F, Dever TE (2005). Mechanistic link between PKR dimerization, autophosphorylation, and eIF2α substrate recognition. Cell.

[8] Launer-Felty K, Wong CJ, Cole JL (2015). Structural analysis of adenovirus VAI RNA defines the mechanism of inhibition of PKR. Biophys J.

[9] Ben-Asouli Y, Banai Y, Pel-Or Y, Shir A, Kaempfer R (2002). Human interferon-γ mRNA autoregulates its translation through a pseudoknot that activates the interferon-inducible protein kinase PKR. Cell.

[10] Namer LS, Osman F, Banai Y, Masquida B, Jung R, Kaempfer R (2017). An ancient pseudoknot in TNF-*α* pre-mRNA activates PKR, inducing eIF2α phosphorylation that potently enhances splicing. Cell Rep.

[11] Cohen-Chalamish S, Hasson A, Weinberg D, Namer LS, Banai Y, Osman F, Kaempfer R (2009). Dynamic refolding of IFN-γ mRNA enables it to function as PKR activator and translation template. Nat Chem Biol.

[12] Jarrous N, Osman F, Kaempfer R (1996). 2-Aminopurine selectively inhibits splicing of tumor necrosis factor alpha mRNA. Mol Cell Biol.

[13] Osman F, Jarrous N, Ben-Asouli Y, Kaempfer R (1999). A cis-acting element in the 3′-untranslated region of human TNF-*α* mRNA renders splicing dependent on the activation of protein kinase PKR. Genes Dev.

[14] Ilan L, Osman F, Namer LS, Eliahu E, Cohen-Chalamish S, Ben-Asouli Y, Banai Y, Kaempfer R (2017). PKR activation and eIF2α phosphorylation mediate human globin mRNA splicing at spliceosome assembly. Cell Res.

[15] Tan SL, Tareen SU, Melville MW, Blakely CM, Katze MG (2002). The direct binding of the catalytic subunit of protein phosphatase 1 to the PKR protein kinase is necessary but not sufficient for inactivation and disruption of enzyme dimer formation. J Biol Chem.

[16] Boyce M, Bryant KF, Jousse C, Long K, Harding HP, Scheuner D, Kaufman RJ, Ma D, Coen DM, Ron D, Yuan J (2005). A selective inhibitor of eIF2α dephosphorylation protects cells from ER stress. Science.

[17] Tsaytler P, Harding HP, Ron D, Bertolotti A (2011). Selective inhibition of a regulatory subunit of protein phosphatase 1 restores proteostasis. Science.

[18] Edery I, Petryshyn R, Sonenberg N (1989). Activation of double-stranded RNA-dependent kinase (dsl) by the TAR region of HIV-1 mRNA: a novel translational control mechanism. Cell.

[19] Ben-Asouli Y, Banai Y, Hauser H, Kaempfer R (2000). Recognition of 5'-terminal TAR structure in human immunodeficiency virus-1 mRNA by eukaryotic translation initiation factor 2. Nucleic Acids Res.

[20] Peden K, Emerman M, Montagnier L (1991). Changes in growth properties on passage in tissue culture of viruses derived from infectious molecular clones of HIV-1LAI, HIV-1MAL, and HIV-1ELI. Virology.

[21] Das AT, Zhou X, Vink M, Klaver B, Verhoef K, Marzio G, Berkhout B (2004). Viral evolution as a tool to improve the tetracycline-regulated gene expression system. J Biol Chem.

[22] Das AT, Harwig A, Vrolijk MM, Berkhout B (2007). The TAR hairpin of human immunodeficiency virus type 1 can be deleted when not required for Tat-mediated activation of transcription. J Virol.

[23] Romano PR, Zhang F, Tan SL, Garcia-Barrio MT, Katze MG, Dever TE, Hinnebusch AG (1998). Inhibition of the double-stranded RNA-dependent protein kinase PKR by Vaccinia virus E3: role of complex formation and the E3 N-terminal domain. Mol Cell Biol.

[24] Novoa I, Zeng H, Harding HP, Ron D (2001). Feedback inhibition of the unfolded protein response by GADD34-mediated dephosphorylation of eIF2α. J Cell Biol.

[25] Jammi NV, Whitby LR, Beal PA (2003). Small molecule inhibitors of the RNA-dependent protein kinase. Biochem Biophys Res Commun.

[26] Lu B, Nakamura T, Inouye K, Li J, Tang Y, Lundbäck P, Valdes-Ferrer SI, Olofsson PS, Kalb T, Roth J, Zou Y, Erlandsson-Harris H, Yang H, Ting JP, Wang H, Andersson U, Antoine DJ, Chavan SS, Hotamisligil GS, Tracey KJ (2012). Novel role of PKR in inflammasome activation and HMGB1 release. Nature.

[27] Schümann M, Gantke T, Mühlberger E (2009). Ebola virus VP35 antagonizes PKR activity through its C-terminal interferon inhibitory domain. J Virol.

[28] Guerrier-Takada C, Eder PS, Gopalan V, Altman S (2002). Purification and characterization of Rpp25 an RNA-binding protein subunit of human ribonuclease P. RNA.

[29] Circle DA, Neel OD, Robertson HD, Clarke PA, Mathews MB (1997). Surprising specificity of PKR binding to delta agent genomic RNA. RNA.

[30] Purcell DF, Martin MA (1993). Alternative splicing of human immunodeficiency virus type mRNA modulates viral protein expression, replication, and infectivity. J Virol.

[31] Emery A, Zhou S, Pollom E, Swanstrom R (2017). Characterizing HIV-1 splicing by using next-generation sequencing. J Virol.

[32] Karn J, Stoltzfus CM (2012). Transcriptional and posttranscriptional regulation of HIV-1 gene expression. Cold Spring Harb Perspect Med.

[33] Jablonski JA, Amelio AL, Giacca M, Caputi M (2010). The transcriptional transactivator Tat selectively regulates viral splicing. Nucleic Acids Res.

[34] Mueller N, Pasternak AO, Klaver B, Cornelissen M, Berkhout B, Das AT (2018). The HIV-1 Tat protein enhances splicing at the major splice donor site. J Virol.

[35] Fischer U, Huber J, Boelens WC, Mattaj IW, Lührmann R (1995). The HIV-1 Rev activation domain is a nuclear export signal that accesses an export pathway used by specific cellular RNAs. Cell.

[36] Yedavalli VS, Neuveut C, Chi YH, Kleiman L, Jeang KT (2004). Requirement of DDX3 DEAD box RNA helicase for HIV-1 Rev-RRE export function. Cell.

[37] Feng Z, Cerveny M, Yan Z, He B (2007). The VP35 protein of Ebola virus inhibits the antiviral effect mediated by double-stranded RNA-dependent protein kinase PKR. J Virol.

[38] Nallagatla SR, Toroney R, Bevilacqua PC (2011). Regulation of innate immunity through RNA structure and the protein kinase PKR. Curr Opin Struct Biol.

[39] Srivastava SP, Kumar KU, Kaufman RJ (1998). Phosphorylation of eukaryotic translation initiation factor 2 mediates apoptosis in response to activation of the double-stranded RNA-dependent protein kinase. J Biol Chem.

[40] Wats JM, Dang KK, Gorelick RJ, Leonard CW, Bess JW, Swanstrom R, Burch CL, Weeks KM (2009). Architecture and secondary structure of an entire HIV-1 RNA genome. Nature.

[41] Tomezsko PJ, Corbin VDA, Gupta P, Swaminathan H, Glasgow M, Persad S, Edwards MD, Mcintosh L, Papenfuss AT, Emery A, Swanstrom R, Zang T, Lan TCT, Bieniasz P, Kuritzkes DR, Tsibris A, Rouskin S (2020). Determination of RNA structural diversity and its role in HIV-1 RNA splicing. Nature.

[42] D'arc M, Ayouba A, Esteban A, Learn GH, Boué V, Liegeois F, Etienne L, Tagg N, Leendertz FH, Boesch C, Madinda NF, Robbins MM, Gray M, Cournil A, Ooms M, Letko M, Simon VA, Sharp PM, Hahn BH, Delaporte E, Mpoudi Ngole E, Peeters M (2015). Origin of the HIV-1 group O epidemic in western lowland gorillas. Proc Natl Acad Sci U S A.

[43] Kuiken C, Korber B, Shafer RW (2003). HIV sequence databases. AIDS Rev.

[44] Berkhout B (1992). Structural features in TAR RNA of human and simian immunodeficiency viruses: a phylogenetic analysis. Nucleic Acids Res.

[45] Jeffrey IW, Kadereit S, Meurs EF, Metzger T, Bachmann M, Schwemmle M, Hovanessian AG, Clemens MJ (1995). Nuclear localization of the interferon-inducible protein kinase PKR in human cells and transfected mouse cells. Exp Cell Res.

[46] Jeffreys AJ, Flavell RA (1977). The rabbit beta-globin gene contains a large insert in the coding sequence. Cell.

[47] Kinniburgh AJ, Mertz JE, Ross J (1978). The precursor of mouse beta-globin messenger RNA contains two intervening RNA sequences. Cell.

[48] Kaempfer R, Ilan L, Cohen-Chalamish S, Turgeman O, Namer LS, Osman F (2019). Control of mRNA splicing by intragenic RNA activators of stress signaling: potential implications for human disease. Front Genet.

[49] Kao SY, Calman AF, Luciw PA, Peterlin BM (1987). Anti-termination of transcription within the long terminal repeat of HIV-1 by tat gene product. Nature.

[50] Berkhout B, Gatignol A, Rabson AB, Jeang KT (1990). TAR-independent activation of the HIV-1 LTR: evidence that Tat requires specific regions of the promoter. Cell.

[51] Selby MJ, Peterlin BM (1990). Trans-activation by HIV-1 Tat via a heterologous RNA binding protein. Cell.

[52] Gatignol A, Buckler-White A, Berkhout B, Jeang KT (1991). Characterization of a human TAR RNA-binding protein that activates the HIV-1 LTR. Science.

[53] Abbink TE, Berkhout B (2008). RNA structure modulates splicing efficiency at the human immunodeficiency virus type 1 major splice donor. J Virol.

[54] Mueller N, van Bel N, Berkhout B, Das AT (2014). HIV-1 splicing at the major splice donor site is restricted by RNA structure. Virology.

[55] Mueller N, Klaver B, Berkhout B, Das AT (2015). Human immunodeficiency virus type 1 splicing at the major splice donor site is controlled by highly conserved RNA sequence and structural elements. J Gen Virol.

[56] Clerzius G, Gélinas J, Gatignol A (2011). Multiple levels of PKR inhibition during HIV-1 replication. Rev Med Virol.

[57] Vrolijk MM, Harwig A, Berkhout B, Das AT (2009). Destabilization of the TAR hairpin leads to extension of the polyA hairpin and inhibition of HIV-1 polyadenylation. Retrovirology.

[58] Ruijter JM, Thygesen HH, Schoneveld OJ, Das AT, Berkhout B, Lamers WH (2006). Factor correction as a tool to eliminate between-session variation in replicate experiments: application to molecular biology and retrovirology. Retrovirology.

[59] van Opijnen T, Kamoschinski J, Jeeninga RE, Berkhout B (2004). The human immunodeficiency virus type 1 promoter contains a CATA box instead of a TATA box for optimal transcription and replication. J Virol.

