# Supplementary material for: HIV co-opts a cellular antiviral mechanism, activation of stress kinase PKR by its RNA, to enable splicing of rev/tat mRNA
Source: Cell Biosci. 2023 Feb 11;13:28. doi: 10.1186/s13578-023-00972-1 (PMC9922466; doi:10.1186/s13578-023-00972-1)
Supplement: Supplementary file 1 — Additional file 1: Fig. S1. PKR inhibitor impairs expression of all size classes of HIV mRNA. A Representative mRNA species from each mRNA class in Fig. 1C (unspliced, 9 Kb; singly spliced, 4 Kb; multiply spliced, 2 Kb) are denoted by red asterisks. The shift in spliced mRNA banding pattern between the HIV-1 wt and the HIV-rtTA variants (TARm and ΔTAR) is caused by sequence differences (rtTA replaced Nef, different 3’UTR). B The denoted species from each mRNA class were quantified using ImageJ software (https://imagej.nih.gov/) (PKRi, μM). Fig. S2. Chemical reactivity of HIV pseudoknot stems P1 and P2. Secondary structure upstream of TAR and lower part of the TAR stem are shown for NL4-3 HIV-1 RNA. G labeled (*) is C in HIV-1 LAI. Chemical reactivity by SHAPE is shown, colors denote reactivity as high (red), moderate (orange), low (green) and little or none (black) [40]. [file 13578_2023_972_MOESM1_ESM.pdf]

Additional file 1

HIV co-opts a cellular antiviral mechanism, activation of stress kinase PKR by its RNA, to enable splicing of *rev/tat* mRNA

Lise Sarah Namer[1], Alex Harwig[2], Stephan P. Heynen[2], Atze T. Das[2], Ben Berkhout[2], and Raymond Kaempfer[1,*]

[1]Department of Biochemistry and Molecular Biology, Institute of Medical Research Israel-Canada, The Hebrew University-Hadassah Medical School, Jerusalem 9112102, Israel

[2]Laboratory of Experimental Virology, Department of Medical Microbiology, Amsterdam UMC, 1105 AZ Amsterdam, The Netherlands

[*]Correspondence:

Raymond Kaempfer, PhD
Dr. Philip M. Marcus Professor of Molecular Biology and Cancer Research
Department of Biochemistry and Molecular Biology
Faculty of Medicine
The Hebrew University
9112102 Jerusalem, Israel
Phone: +9722-6758389
Email: kaempfer@hebrew.edu

Contents

Supplementary Figures

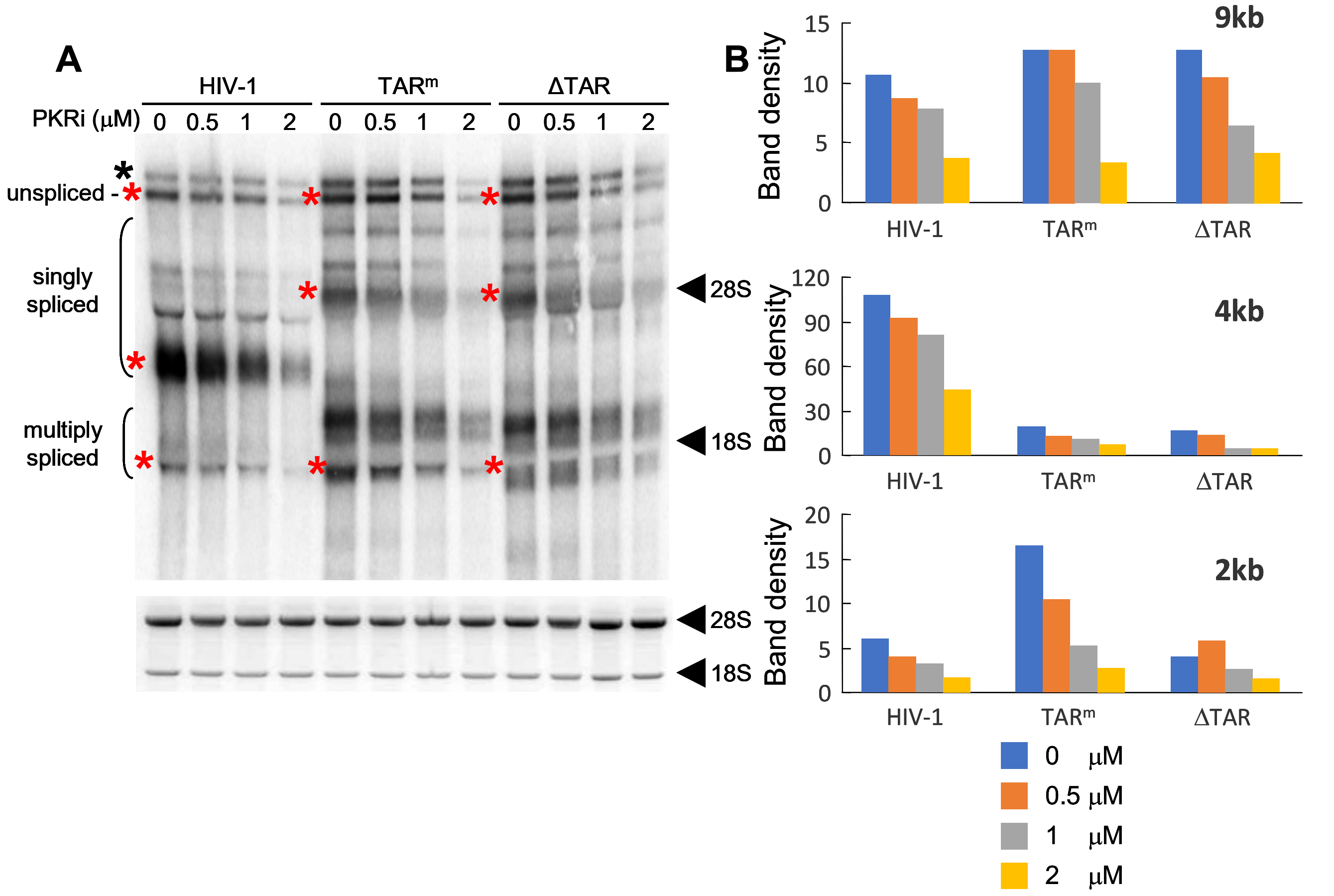

Additional file 1: Fig. S1 PKR inhibitor impairs expression of all size classes of HIV mRNA. **A** Representative mRNA species from each mRNA class in Fig. 1C (unspliced, 9 Kb; singly spliced, 4 Kb; multiply spliced, 2 Kb) are denoted by red asterisks. The shift in spliced mRNA banding pattern between the HIV-1 wt and the HIV-rtTA variants (TAR^{m} and ΔTAR) is caused by sequence differences (rtTA replaced Nef, different 3'UTR). **B** The denoted species from each mRNA class were quantified using ImageJ software (https://imagej.nih.gov/) (PKRi, μM).

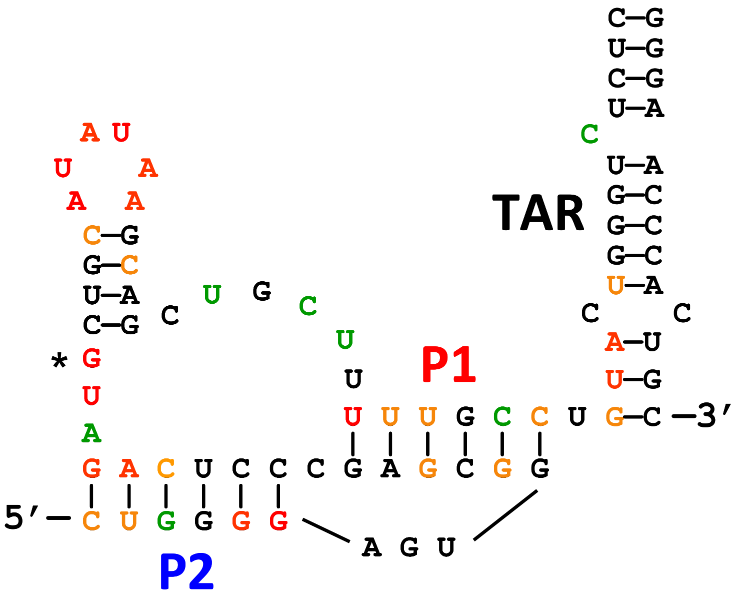

Additional file 1: Fig. S2 Chemical reactivity of HIV pseudoknot stems P1 and P2. Secondary structure upstream of TAR and lower part of the TAR stem are shown for NL4-3 HIV-1 RNA. G labeled (*) is C in HIV-1 LAI. Chemical reactivity by SHAPE is shown, colors denote reactivity as high (red), moderate (orange), low (green) and little or none (black) [40].
